# Head in the clouds: Re-imagining the experimental laboratory record for the web-based networked world

**DOI:** 10.1186/1759-4499-1-3

**Published:** 2009-10-29

**Authors:** Cameron Neylon

**Affiliations:** 1 STFC Rutherford Appleton Laboratory, Harwell Science and Innovation Campus, Didcot, UK

## Abstract

The means we use to record the process of carrying out research remains tied to the concept of a paginated paper notebook despite the advances over the past decade in web based communication and publication tools. The development of these tools offers an opportunity to re-imagine what the laboratory record would look like if it were re-built in a web-native form. In this paper I describe a distributed approach to the laboratory record based which uses the most appropriate tool available to house and publish each specific object created during the research process, whether they be a physical sample, a digital data object, or the record of how one was created from another. I propose that the web-native laboratory record would act as a feed of relationships between these items. This approach can be seen as complementary to, rather than competitive with, integrative approaches that aim to aggregate relevant objects together to describe knowledge. The potential for the recent announcement of the Google Wave protocol to have a significant impact on realizing this vision is discussed along with the issues of security and provenance that are raised by such an approach.

## Introduction

Automated experimentation brings the promise of a much improved record of the research process. Where experiments are sufficiently well defined that they can be carried out by automated instrumentation or computational resources it is to be expected that an excellent record of process can and will be created. In "Big Science" projects from particle physics [1] to genome sequencing [2] the sharing of records about samples and objects, experimental conditions and outputs, and the processing of data is a central part of planning and infrastructure, and often a central part of justifying the investment of resources. As some segments biological science have become industrialized with greater emphasis on high throughput analysis and the generation of large quantities of data sophisticated systems have been developed to track the experimental process and to describe and codify the results of experiments through controlled vocabularies, minimal description standards [3], and ontologies [4].

None of this has had a major impact on the recording process applied to the vast majority of research experiments, which are still carried out by single people or small teams in relative isolation from other research groups. The vast majority of academic research is still recorded in paper notebooks and even in industry the adoption of electronic recording systems is relatively recent and remains patchy. A paper notebook remains a means of planning and recording experiments that is both flexible, comfortable to use, and has a long history of successful use. However, it is starting to fail as an effective means of recording, collating, and sharing data due to the increasing volume and changing nature of the data that researchers are generating, The majority of data generated today is born digital. The proportion of global data generated in 2002 that was recorded on hard disks was estimated at over 90% of a total of around five exabytes with print accounting for less than 0.05% of the total [5]. In the case of small laboratory data some printouts make it into bound notebooks. In most cases however, data remains distributed on a collection of laboratory and personal hard disks. The record of data analysis, the conversion of that digital data into new digital objects and finally into scientific conclusions is, in most cases, poorly recorded.

The question of reproducibility lies at the heart of scientific method and there are serious concerns that much currently published science is of limited value due to poor record keeping. Data sharing mandates from research funders are driven, at least in part, by a concern about reproducibility. Opposition to those mandates is driven to a significant extent by concerns of the value of sharing data that cannot be placed in context due to inadequate recording of its production. With digital instrumentation, more complex experiments, and data volumes increasing a paper based record is now longer capable of providing the necessary context.

It is noteworthy in this context that a number of groups have felt it necessary to take an active advocacy position in trying to encourage the wider community that the reproducibility of data analysis is a requirement, and not an added bonus [6,7]. The promise of digital recording of the research process is that it can create a reliable record that would support automated reproduction and critical analysis of research results. The challenge is that the tools for generating these digital records must outperform a paper notebook while simultaneously providing enough advanced and novel functionality to convince users of the value of switching

At the same time the current low level of adoption means that that field is wide open for a radical re-imagining of how the record of research can be created and used. It lets us think deeply about what value the different elements of that record have for use and re-use and to take inspiration from the wide variety of web-based data and object management tools that have been developed for the mass consumer market. This paper will describe a new way of thinking about the research record that is rooted in the way that the World Wide Web works and consider the design patterns that will most effectively utilize existing and future infrastructure to provide a useful and effective record.

### The distinction between capturing process and describing an experiment

In discussing tools and services for recording the process of research there is a crucial distinction to be made between capturing a record of as it happens and describing an experiment after the event. There is an important distinction between data, the raw material produced by an experiment, including the record of that experiment; information, which places that data in a context that allows inferences and deductions to be made; creating knowledge. A large part of the tension between researchers who develop systems for describing knowledge in structured form and research scientists who need a record of the processes carried out in the laboratory derives from a misunderstanding about whether data, information, or knowledge is being recorded. The best way to maximise success in recording the important details of a research process is to capture the data and record as they are generated, or in the case of plans, before they are generated. However, most controlled vocabularies and description systems are built, whether explicitly or implicitly, with the intention of describing the knowledge that is inferred from a set of experiments, after the results have been considered. This is seen mostly clearly in ontologies that place a hypothesis at the core of the descriptive structure or assume that the "experiment" is a clearly defined entity before it has been carried out.

These approaches work well for the highly controlled, indeed, industrialised studies that they were generally designed around. However they tend to fail when applied to small scale and individual research, and particularly in the situations where someone is "trying something out". Most of the efforts to provide structured descriptions of the research process start with the concept of an "experiment" that is designed to test a "hypothesis" (see e.g [8,9]). However in the laboratory the concept of "the hypothesis" very often doesn't usefully apply to the detail of the experimental steps that need to be recorded. And the details of where a specific experiment starts and finishes are often dependent on the viewer, the state of the research, or the choices made in how to publish and present that research after the fact. Products or processes may be part of multiple projects or may be later used in multiple projects. A story will be constructed later, out of these elements, to write a paper or submit a database entry but at the time the elements of this story are captured the framework may be vague or non-existent. Unexpected results clearly do not fit into an existing framework but can be the launching point for a whole new programme. The challenge therefore is to capture the elements of the research process in such a way that the sophisticated and powerful tools developed for structured description of knowledge can be readily applied once the story starts to take form. That is the data and record should be captured in such a way that it can easily be placed in context to provide information, which in turn can be structured and presented as new knowledge.

## The Web Native Lab Notebook

If we are to consider a web-native approach to capturing the scientific record we need first to consider the laboratory notebook. The lab notebook is, at its core, a journal of events, an episodic record containing dates, times, bits and pieces of often disparate material, cut and pasted into a paper notebook. There are strong analogies between this view of the lab notebook as a journal and the functionality of Web logs or "Blogs". Blogs contain posts which are dated, usually linked to a single author, and may contain embedded digital objects such as images or videos, or indeed graphs and charts generated from online datasets as well as free or structured text. Each post has an address on the web, given by a post number or title (or both). Thus a Blog provides much of the functionality of a laboratory notebook: it feels like a journal and it can contain and present both free text and structured text such as tables. While it may not have page or volume numbers each post will have its own address on the web, a URL that points uniquely at that one piece of the record, and can be passed to collaborators to share specific objects or be used to index specific protocols, experiments, samples, or pieces of data.

### A "semantic web ready" laboratory record

The creation of individually addressable objects is crucial because it enables these objects, whether they are datasets, protocols, or pointers to physical objects such as samples, to play a part in the semantic web [10]. The root concept of the semantic web is that the relationships between objects can be encoded and described. For this to be possible those objects must be uniquely addressable resources on the web. By creating individual posts or pages the researcher is creating these individual resources; and again these can represent physical objects, processes, or data. It is possible to describe the relationships between these resources via sophisticated semantic tools such as the Resource Description Framework (RDF) or locally via statements within the posts. However it not necessary to take these approaches as it is also possible to simply express relationships that directly leverages the existing toolset on the web is by linking posts together.

### Feeds change the lab notebook from a personal record to a collaborative document

The other key functionality of the web to focus on is that of the "feed". Feeds, whether they are RSS or Atom are XML documents that are regularly updated providing a stream of "events" which can then be consumed by various readers, Google Reader being one of the most popular. Along with the idea of hyperlinks between objects the feed provides the crucial difference between the paper based and web-native lab notebook. A paper notebook (whether it is a physical object or "electronic paper") is a personal record. The web-native lab notebook is a collaborative notification tool that announces when something has happened, when a sample has been created, or a piece of data analysed.

**Figure 1:**
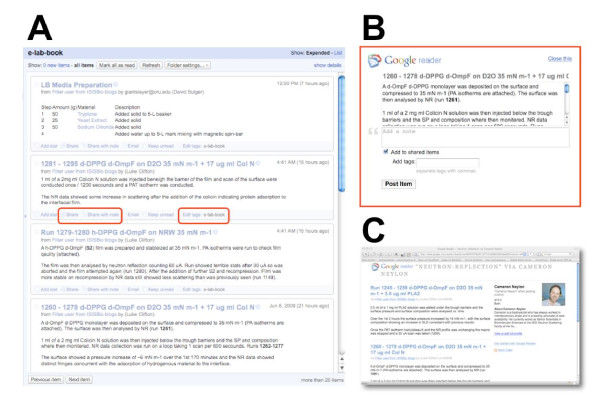
**Using feeds and feed readers to aggregate and push laboratory records**. **A**) A screenshot of Google Reader showing an aggregated feed of laboratory notebook entries from http://biolab.isis.rl.ac.uk. Two buttons are highlighted which enable "sharing" to anybody who follows the user's feed or adding a tag. **B**) Sharing can also include annotating the entry with further information or tagging the entry to place it in a specific category. **C**) A tag can create a new feed. In this panel a feed has been created that contains all items tagged "neutron reflection". The feed for appriate tags can be consumed by readers with a specific interest such as collaborators on a specific project, regulatory agencies wishing to monitor specific classes of experiment, or funders who require access to research summaries.

Despite of the historical tendency to isolated research groups discussed above, these independent groups *are *banding together as research funders demand larger coordinated projects. Tasks are divided up by expertise and in many cases also divided geographically between groups that have in the past probably not even had good internal communication systems. Rapid and effective communication between groups on the details of ongoing projects is becoming more and more important and is increasingly a serious deficiency in the management of these collaborations. In addition reporting back to sponsors via formal reports is an increasing burden. The notification systems enabled via the generation of feeds go a significant way towards providing a means of dealing with these issues. Within a group the use of feeds and feed readers can provide an extremely effective means of pushing information to those who need to either track or interact with it. In addition the idea of *selectively *pushing *specific *elements of the record of interest to a specific group could also be adopted to push either raw productivity data or a much smaller subset of items, including summaries, of interest to funding agencies (Figure 1). The web native lab notebook should bring the collaborative authoring and discussion tools provided by the read-write web to bear on the problem of communicating research results.

### Integrating tools and services

With the general concept of the record as a blog in place, enabling us to create a set of individually addressable objects, and link them together, as well as providing feeds describing the creation of these objects, we can consider what tools and services we need to author and to interact with these objects. Again blogs provide a good model here as many widely used authoring tools can be used directly to create documents and publish them to blog systems. Tools based on the Atom Publishing Protocol [11,12] can hide the complications of publishing documents to the web from the user and made it easy to develop sophisticated services that can push content from one place to another online. Recent versions of Microsoft Office include the option of publishing documents to online services and a wide range of web services now make it easy to push content from wordprocessors, mobile phones, email, or any connected source to virtually any other.

A wide variety of web based tools and plugins are available to make the creation and linking of blog posts easy. Particularly noteworthy are tools such as Zemanta, a plugin which automatically suggests appropriate links for concepts within a post [13]. Zemanta scans the text of a post and identifies company names, concepts that are described in Wikipedia and other online information sources, using an online database that is built up from the links created by other users of the plugin. The service suggests possible links and tags to the users, and then exploits the response of the user to those suggestions to refine the model for future suggestions.

Sophisticated semantic authoring tools such as the Integrated Content Environment (ICE) developed at the University of Southern Queensland [14,15] provide a means of directly authoring semantic documents that can then be published to the web. ICE can also be configured to incorporate domain specific semantic objects that generate rich media representations such as three dimensional molecular models. These tools are rapidly become very powerful and highly useable, and will play an important role in the future by making rich document authoring straightforward.

### Where do we put the data?

**Figure 2:**
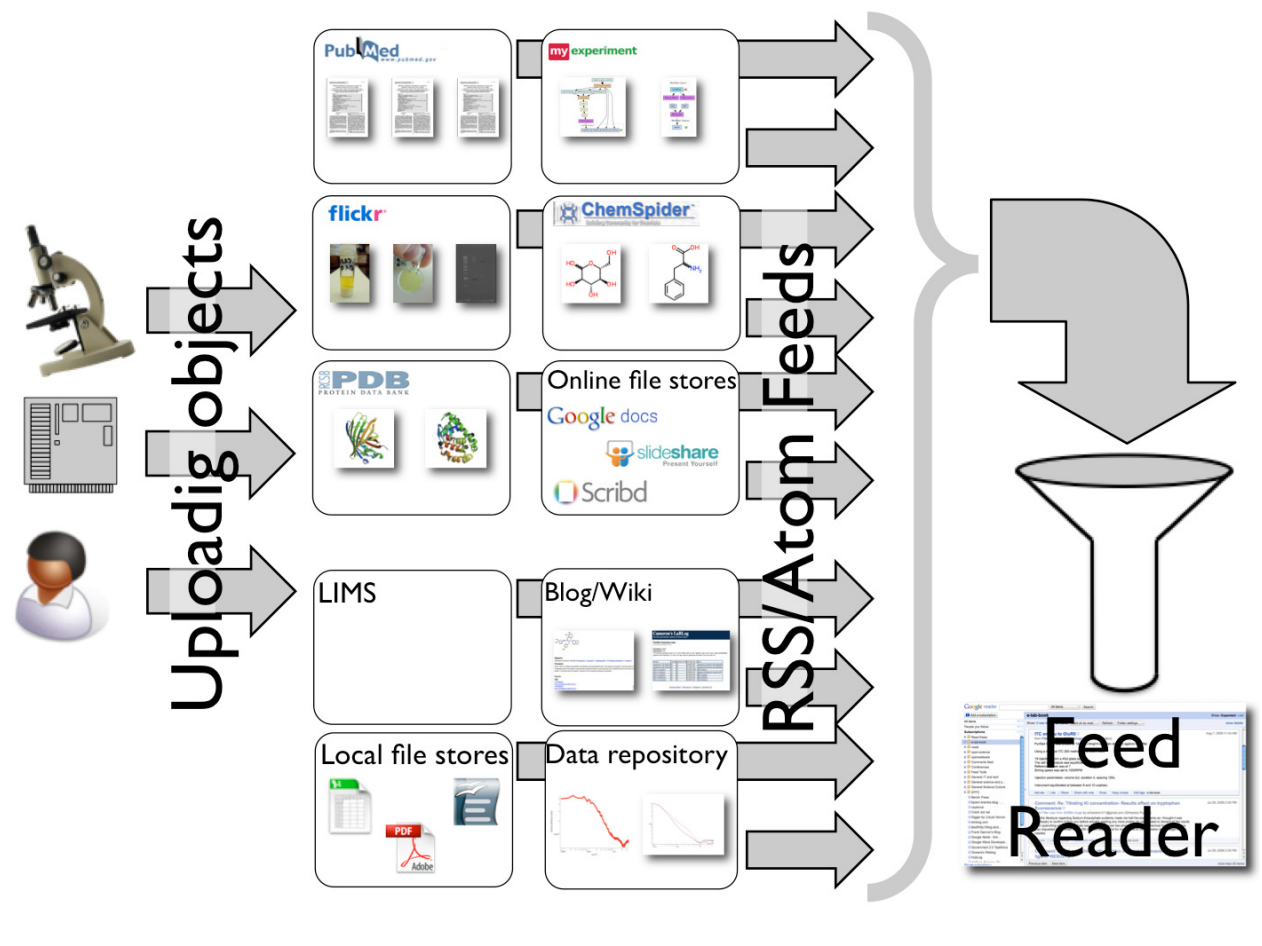
**Distributing research objects to online services and re-aggregating them via feeds**. Researchers, instruments, or computers may create digital objects such as data, workflows, descriptions, and presentations as well as references to physical objects such as samples and materials. These objects can be stored in a wide range of services that provide specific functionality. Using feeds the record of these depositions can be re-aggregated, tracked, and processed using standard tools such as Feed readers.

With the authoring of documents in hand we can consider the appropriate way of handling data files. At first sight it may seem simplest to upload data files and embed them directly in blog posts. However, the model of the blog points us in a different direction here again. On a blog images and video are not generally uploaded directly, they are hosted on an appropriate, specialised, external service and then embedded them on the blog page. Issues about managing the content and providing a highly user-friendly viewer are handled by the external data service. Hosting services are optimized for handling specific types of conten; Flickr for photos, YouTube (or Viddler or Bioscreencast) for video, Slideshare for presentations, Scribd for documents (Figure 2).

While it might be argued that the development of these specialist services and embedding capabilities grew out of deficiencies in generic hosting platforms it is also true that these specialist platforms have exploited the economies of scale that arise from handling similar content together. Scientific infrastructure is resource limited and there is a strong argument that rather than building specialist publishing platforms it is more effective to use generic platforms for publishing. Specialist datahandling services can then grow up around specific data types and benefit from the economies of scale that arise from aggregating types together. In an ideal world there would be a trustworthy data hosting service, optimized for your specific type of data, that would provide cut and paste embed codes providing the appropriate visualizations in the same way that videos from YouTube can easily be embedded.

Some elements of these services exist for research data. Trusted repositories exist for structural data, for gene and protein sequences, and for chemical information. Large-scale projects are often required to put a specific repository infrastructure in place to make the data they generate available. And in most cases it is possible to provide a stable URL which points at a specific data item or dataset. It is therefore possible in many cases to provide a link directly to a dataservice that places a specific dataset in context and can be relied on to have some level of curation or quality control and provide additional functionality appropriate to the datatype. Currently many of these URLs encode database queries rather than providing a direct link. To be most effective and reliable such URLs need to be "Cool" [16]. That is they should be stable, human readable, and direct addresses rather than queries. Query engines may change, and database schemas may be modified, but the address of the underlying objects needs to stay constant for the linked data web to be stable enough to form.

What is less prevalent is the type of embedding functionality provided by many consumer data repository services. ChemSpider http://www.chemspider.com is one example of a service that does enable the embedding of both molecules and spectra into external web pages. This is still clearly an area for development and there are discussions to be had about both the behind the scenes implementation of these services as well as the user experience but it is clear that this kind of functionality could play a useful role in helping researchers to connect information on the web up. If multiple researchers use the ChemSpider molecule embedding service to reference a specific molecule then all of those separate documents can be unambiguously assigned as describing the same molecule. This linking up of individual objects through shared identifiers is precisely what gives the semantic web its potential power.

A more general question is the extent to which such repositories can or will be provided and supported for less common data types. The long term funding of such data repositories is at best uncertain and at worst non-existent. Institutional repositories are starting to play a role in data archiving and some research funders are showing an interest. However there is currently little or no coordinated response to the problem of how to deal with archiving data in general. Piecemeal solutions and local archiving are likely to play a significant role. This does not necessarily make the vision of linked data impossible, all that is required is that the data be placed somewhere where it can be referenced via a URL. However, to enable rich functionality to manipulate and visualize that data it will be necessary to find funding sources and business models that can support and enable the development of high quality data repositories. In our model of the Blog as a lab notebook a piece of data can be uploaded directly to a post within the blog. This provides the URL for the data, but will not in and of itself enable visualization or manipulation. Nonetheless the data will remain accessible and addressable in this form. We can take a step forward by simply putting it on the web but to enable other researchers to use those objects most effectively it will be important to provide rich functionality. This will be best supported via provision in centralised services where economies of scale can be found.

A key benefit of this way of thinking about the laboratory record is that items can be distributed in many places depending on what is appropriate. It also means is that the search mechanisms we use to find objects and information on the web to index and search our own laboratory material. Web search relies primarily on mechanisms like Page Rank that prioritise how specific addresses are linked in to the wider web. By linking our record into that wide web we enable Google and other search engines to identify our most important datasets, based on how they are connected to the rest of our research record as well as to the wider research effort.

This is a lightweight way of starting to build up a web of data. It doesn't provide the full semantic power of the linked data web as envisioned by Tim Berners-Lee and others but it also doesn't hold the same challenges and fears for users. If we can get data up on the web and identify relationships between them it doesn't matter so much to start with whether these relationships are fully described as long as there is enough contextual data to make it useful. Tagging or key-value pairs using the tools that are already available and more widely adopted by the general user community would enable us to make a good start on improving data availability and discoverability while the tools to provide more detailed semantic markup are developed.

However while distribution has benefits, it also poses significant risks. Services can fail, links can and do break, and interoperability is made more complex and can easily be compromised by developments of one service that are not mirrored on another. It would also seem at first sight to be opposed to integrative approaches that aggregate related objects together. However, such approaches, inspired by the "Datument" concept of Rzepa and Murray-Rust [17], can be more properly seen as providing the opportunity to aggregate, contain, and represent *knowledge *once it has been generated from the raw material. Our aim in distributing the elements of the record, the raw *data*, is therefore to provide the contextual *information *either through links, or through metadata, to make it straightforward to aggregate those elements into datuments for the presentation, publishing, and archival of *knowledge*.

### Distributed sample logging systems

The same logic of distributing data according to where it is most appropriate to store it can also be applied to the recording of samples. In many cases, tools such as Laboratory Information Management System (LIMS) or sample databases will already be in place. In most cases these are likely to applied to a specific subset of the physical objects being handled; a LIMS for analytical samples, a spreadsheet for oligonucleotides, and a local database, often derived from a card index, for lab chemicals? As long as it is possible to point to the record for each physical object independently with the required precision you need then these systems can be used directly. Although a local spreadsheet may not be addressable at the level of individual rows Google Spreadsheets can be addressed in this way. Individual cells can be addressed via a URL for each cell and there is a powerful API that makes it possible to build services to make the creation of links easy. Web interfaces can provide the means of addressing databases via URL through any web browser or http capable tool.

Samples and chemical can also be represented by a post within a Blog. This provides the key functionality that we desired; a URL endpoint that represents that object. This can provide a flexible approach which may be more suited to small laboratories than heayweight, database backed systems, designed for industry. When samples involve a wide variety of different materials put to different uses, the flexibility of using an open system of posts rather than a database with a defined schema can be helpful.

In many cases it may be appropriate to use multiple different systems, a database for recording oligonucleotides, a spreadsheet for tracking environmental samples, and a full blown LIMS to enable barcoding and monitoring samples through preparation for sequencing. Similar to the data case, it is best to use a system that is designed for or best suited to creating a record for the specific set of samples. These systems are better developed than they are for data - but many of the existing systems don't allow a good way of pointing at the record for specific samples from an external document - and very few make it possible to do this via a simple and cool URI.

### Full distribution of materials, data, and process: The lab notebook as a feed of relationships

At this point it may seem that the core remaining component of the lab notebook is the description of the actions that link material objects and data files the record of process. However even these records could be passed to external services that might be better suited to the job. Procedures are also just documents. Maybe they are text documents, but perhaps they are better expressed as spreadsheets or workflows (or rather the record of running a workflow). These may well be better handled by external services, be they word processors, spreadsheets, or specialist services. They just need to be somewhere where, once again, it is possible to unambiguously point at them.

**Figure 3:**
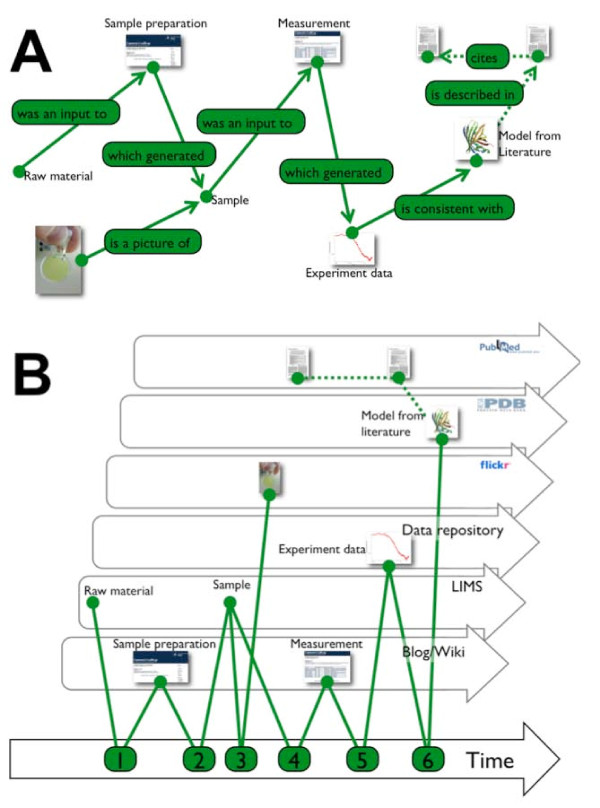
**The lab notebook as a time dependent feed of relationships**. **A**) A series of research objects collected as part of an experiment. A raw material is converted into a sample that is then subjected to analysis. A photo is taken of the sample. The analysis generates data that is in turn is described as consistent with a previously published model. The connection of the new data with the model, and onto the literature that describes the generation of that model wires the new, unpublished experiment, into the wider web. The dotted green lines show previously existing relationships between the model data and published papers. **B**) The same set of objects and relationships shown as a set of time dependent feeds generated by a set of services. Each object is accessible on a feed generated by the service where it is hosted. The "laboratory notebook" is the feed shown at the bottom which records and describes relationships between the research objects.

What we are left with is the links that describe the relationship between materials, data, and process, arranged along a timeline. The laboratory record, the web-native laboratory notebook, is reduced to a feed that describes these relationships; that notifies users when a new relationship is created or captured (Figure 3). This could be a simple feed containing plain hyperlinks or it might be a sophisticated and rich feed that uses one or more formal vocabularies to describe the semantic relationship between items. In principle it is possible to mix both, gaining the best of detailed formal information where it is available but linking in relationships that are less clearly described where possible. That is, this approach can provide a way of building up a linked web of data and objects piece by piece, even when the details of vocabularies are not yet agreed or in place.

## Implementation: Tools and services

At one level this framework of links and objects can be put together out of existing pieces from online tools and services but on another the existing tools are totally inadequate. What is lacking is an integrated framework for managing the wide range of research objects that we create. While there are a wide range of freely accessible sites for hosting data of different or arbitrary types, documents, and bookmarks, and these can be linked together in various ways there are very few tools and services that provide the functionality of managing links within the kind of user friendly environment that would be like to encourage adoption. Most existing web tools have also been built to be "sticky" so as to keep users on the site. This means that they are often not good at providing functionality to link out to objects on other services. A central problem is therefore the lack of good tools to integrate the management of this disparate set of objects.

The linked data web-native notebook described above could potentially be implemented using existing tools if an integration tool or framework could be implemented. A full implementation would involve a variety of structured documents placed on various services that used specific controlled vocabularies to describe them. The relationships between these documents would then be specified in a formal semantic feed such as for example a structured RSS feed generated via a separate integrating service or be available by querying a RDF data store via SPARQL. Either of these approaches would then require a controlled vocabulary or vocabularies to be used in description of relationships and therefore the feed.

In practice, while this is technically feasible, for the average researcher the vocabularies are often not available or not appropriate. The tools for generating semantic documents, whether XML or RDF based are not, where they exist at all, designed with the general user in mind. The average lab is therefore restricted to a piecemeal approach based on existing, often general consumer web services. This approach can go some distance, using wikis, online documents, and data visualization extensions. An example of this approach is described in [18] where a combination of Wikis, GoogleDoc Spreadsheets and visualization tools based on the GoogleChart API were used in a distributed set of pages that linked data representations to basic data to the procedures used to generate it through simple links. However, because these are just simple hypertext links in free text documents this approach currently can't exploit the full potential of a semantic approach. A RDF description of the experiment was created in this case by developing a *specific *parser to convert from the spreadsheet and experimental records to RDF and deposit into a triple store.

Clearly there is a gap in the tool set for delivering a linked experimental record. But what is needed to fill that gap? High quality dataservices are required and are starting to appear in specific areas with a range of business models. Many of those that exist in the consumer space already provide the type of functionality that would be required including RSS feeds, visualization and management tools, tagging and categorization, and embedding capabilities. Slideshare http://www.slideshare.net and Flickr (flickr.com) are excellent models for scientific data repositories in many ways.

Sophisticated collaborative online and offline document authoring tools are available. Online tools include blogs and wikis, and increasingly offline tools including Microsoft Word and Open Office, provide a rich and useable user experience that is well integrated with online publishing systems. Tools such as ICE can provide a sophisticated semantic authoring environment making it possible to effectively link structured information together.

### The missing link

What are missing are the tools that will make it easy to connect items together. Semantic authoring systems solve part of the problem by enabling the creation of structured documents and in some cases by assisting in the creation of links between objects. However these are usually inward looking. The key to the web-native record is that it is integrated into the wider web, monitoring sites and feeds for new objects that may need to be incorporated into the record. These monitoring services would then present these objects to the user within a semantic authoring tool, ideally in a contextual manner.

**Figure 4:**
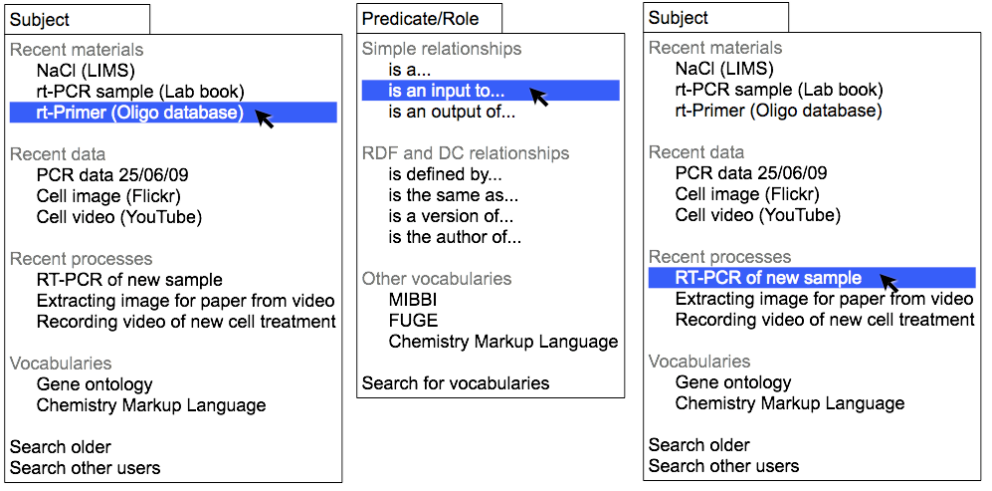
**A simple conceptual tool for connecting web objects together via relationships**. A simple menu based tool for identifying the connections between research objects. A range of web services are monitored to identify new objects, data, processes, documents, or controlled vocabulary terms that may be relevant to the user. In this simple tool these are presented as potential subjects and objects in drop down menus. The relationship can be selected from a central menu. The output of the tool is a feed of relationships between web accessible objects.

The conceptually simplest system would monitor appropriate feeds for new objects and present these to the user as possible inputs and outputs. The user would then select an appropriate set of inputs and outputs and select the relationship between them from a limited set of possible relationships (is an input to, is an output of, generated data). This could be implemented as three drop down menus in its simplest form (Figure 4) but this would only apply after the event. Such a tool would not be particularly useful in planning experiments or as a first pass recording tool and would therefore add another step to the recording process.

### Capturing *in silico *process: A new type of log file

In an ideal world, the whole process of creating the linked record would be done behind the scenes, without requiring the intervention of the user. This is likely to be challenging in the experimental laboratory but is entirely feasible for in silico work and data analysis within defined tools. Many data analysis tools already generate log files, although as data analysis tools have become more GUI driven these have become less obvious to the user and more focussed on aiding in technical support. Within a given data analysis or computational tool objects will be created and operated on by procedures hard coded into the system. The relationships are therefore absolute and defined.

The extensive work of the reproducible research movement on developing approaches and standards to recording and communicating computational procedures has largely focussed on the production of log files and command records (or scripts) that can be used to reproduce an analysis procedure as well as arguing for the necessity to provide running code and the input and intermediate data files. In the linked record world it will be necessary to create one more "logfile" that describes the relationships between all the objects created by reference to some agreed vocabulary. This "relationships logfile" which would ideally be RDF or a similar framework is implicit in a traditional log file or script but by making it explicit it will be possible to wire these computational processes into a wider web of data automatically. Thus the area of computational analysis is where the most rapid gains can be expected to be made as well as where the highest standards are likely to be possible. The challenge of creating similar log files for experimental research is greater, but will benefit significantly from building up experience in the *in silico *world.

### Systems for capturing physical processes

For physical experimentation, where it is more difficult to automatically capture the objects being used and the role they are playing, we need to develop tools that assist the scientist in capturing these connections. We will have feeds of potential materials, samples, and data but require tools that assist the user in authoring the description of process in a way that captures which objects have been used or created and the connections between them. Such an authoring tool would automatically tracks feeds of possible input and output objects and provide these as options as the researcher describes what they are planning or what they are doing. For example the description of an experiment that generates an image would trigger the system to look at recent items from the feed (or feeds) of the appropriate image service(s). Recent options would be presented to the user in a drop down menu for selection. The selection of the correct item would add a link from the document to the image. As the input samples have already been registered, either by logging commercial samples into the lab system, or having been generated automatically as outputs for a previous process typing "sample for treatment A" would trigger the system to link to that record and incorporate a machine readable statement within the document that "sample for treatment A" was the input for the process "an image was taken" which generated data (the image). The objects, including the description of the process, are then linked together, via the outgoing feed by their appearance in one process, making it possible to describe a composite object that is the overall experiment.

Such a system would consist of two parts; first an intelligent feed reader that monitors all the relevant feeds, laboratory management systems for samples, data repositories, or laboratory instruments, for new data. In a world in which it seems natural that the Mars Phoenix lander should have a Twitter account, and indeed the high throughput sequencers at the Sanger Centre send status updates via Twitter, the notion of an instrument automatically providing a status update on the production of data seems natural. What are less prevalent are similar feeds generated by sample and laboratory information management systems although the principles are precisely equivalent; when an object (data or sample or material) is created or added to the system a feed item is created and pushed out for notification.

The second part of the system is more challenging. Providing a means of linking inputs to outputs, via for example drop down menus is relatively straightforward. Even the natural language processing required to automatically recognise where links should be created is feasible with today's technology. But capturing what those links mean is more difficult. There is a balance that needs to be found between providing sufficient flexibility to describe a very wide range of possible connections ("is an input to", "is the data generated from") and providing enough information, via a rigorously designed controlled vocabulary, to enable detailed automated parsing by machines. Where existing vocabularies and ontologies exist and are directly applicable to the problem in hand these should clearly be used and strongly supported by the authoring tools. In the many situations where they are not ideal then more flexibility needs to be supported allowing the researcher to go off the planned track in an unexpected direction. However at the same time ambiguity should be avoided. Thus such a system needs to "nudge" the user into using the most appropriate available structured description of their experiment, while allowing them the choice to use none, but at the same time helping them avoid using terms or descriptions that could be misinterpreted. Not a trivial system to build.

## A wave to wash the problems away?

**Figure 5:**
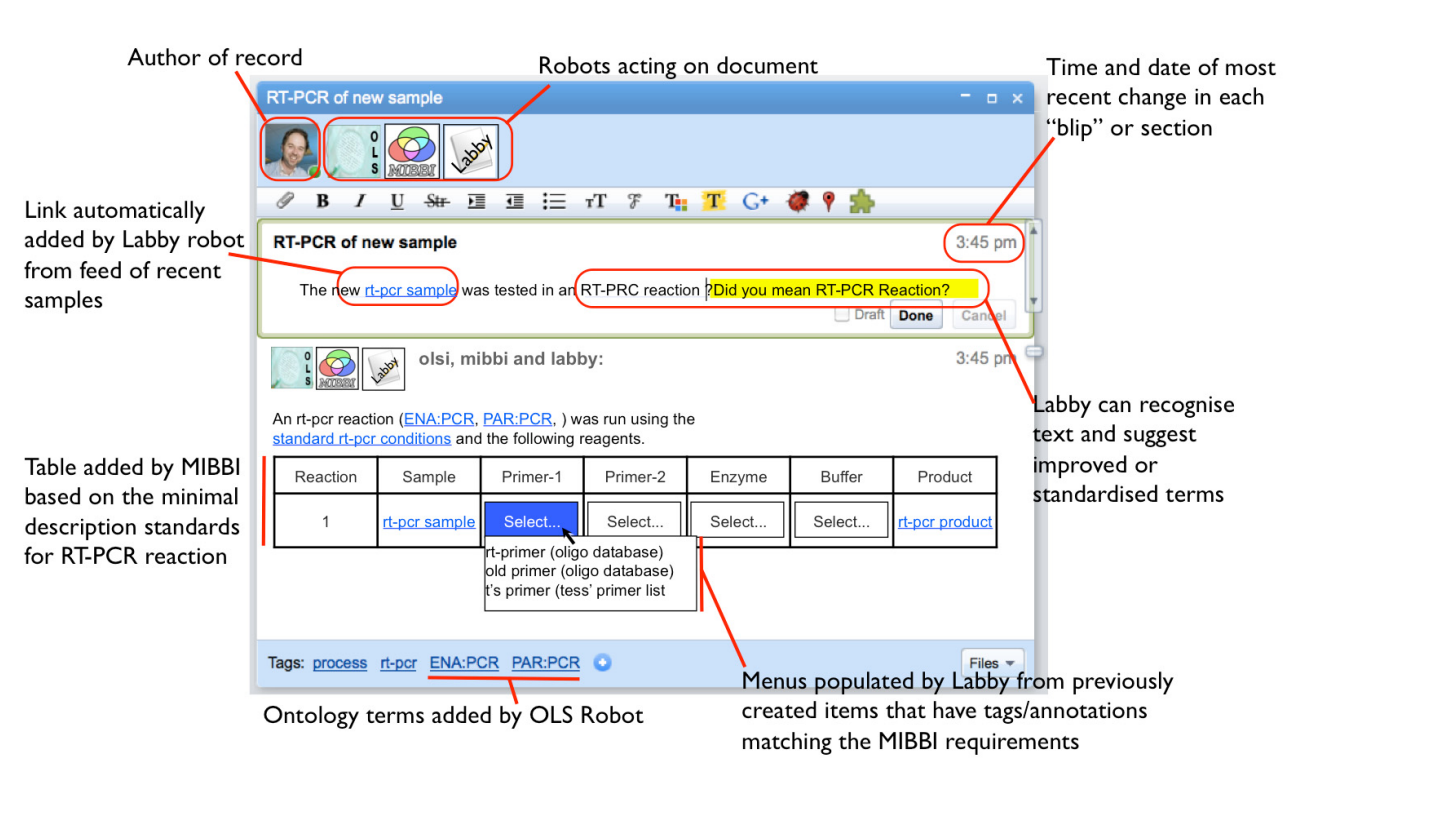
**A mockup of a Google Wave based laboratory recording system**. The document editing window of the current Wave client is shown with four participants, the human author, and three Robots that "read" the document and make automatic changes and annotations. The suggested Robots (which do not currently exist) are "OLSI" a Robot that compares text to terms available via the EBI Ontology Lookup Service, "MIBBI" a Robot that searches for structured minimal description requirements for specific classes of experiment and provides structured means for adding the required information, probably via tables and drop down menus, and Labby. This Robot generates a feed of objects created within the Wave framework and notifies external services. It also aggregates feeds from other services and provides lists of possible objects for particular purposes. In the top panel Labby has recognised a previously described sample ("new rt-pcr sample") and created a link to the appropriate record. Labby has recognized mistyped text and suggested a specific term from a dictionary that is more appropriate. In the bottom panel, after the correction has been accepted, OLSI recognises the term and inserts the appropriate ontology terms. MIBBI has then searched for a structured description format that relates to those ontology terms and inserted a table to aid in capturing the required information. Labby has identified previous Wave objects that have been tagged as "primers" and provided them to the table generated by MIBBI to generate a drop down menu.

The previous discussion identifies three key needs: strong records of process, when and where things happened and how those events relate to each other; a coordination space for managing and integrating diverse feeds of information, and the means of creating those feeds in the first place; and flexible means of suggesting and encouraging the use of structured descriptions and vocabularies while not enforcing them. Some of these issues are also being tackled by technical developments aimed at the wider consumer web, of which Google Wave [19,20] has received the most coverage. In this section I consider the potential of Wave to provide a framework in which the desired functionality can be delivered (Figure 5).

### What is Wave?

Wave is described by Google as "how email would look if it were invented today". To the user it appears initially as a communication platform that combines the asynchronous functionality of email with the synchronous communication of chat or instant messaging. The key difference is rather than being a file that is duplicated and transmitted from place or person to another, as is the case in email, a Wave is an XML document that is housed on a server and updated in real time. This means in addition to acting as a message platform a Wave can also act as a collaborative document, providing some of the functionality of a Wiki. Like a wiki the XML document is versioned and edits are recorded, providing a complete record of all the changes made, satisfying some of our versioning requirements.

The fact that Wave offers real-time versioned collaborative authoring is a valuable additional functionality. However the key technical advance for the current discussion is the way Wave can utilize web services to enhance and modify documents. Wave introduces the idea of Robots; web services that are first class participants in the conversation, with the ability to monitor, interpret, and modify the wave document. It is these Robots and the way they provide the user with an easy connection to the web services behind them that has significant potential to provide a framework that can supply the versioning, feed integration, and vocabulary tools required.

### Robots as feed consumers

The first obvious use of a Wave Robot is as a feed consumer representing a specific instrument or analysis process. Adding the Robot as a participant in a Wave describing and experiment signals that the report is expecting to receive data from the instrument that the Robot represents. The web service behind the Robot will monitor the instrument, perhaps watching for a file to appear in a designated directory, and insert the data, or embed a link to its primary location, into the Wave. The Robot might also search for appropriate metadata within the Wave, such as sample identifiers to connect with the output data. Where the Robot has taken data from the Wave for analysis by an external service this link can be made explicit and recorded within the wave through an annotation or externally in a separate record. By selecting a specific Robot relevant to the specific experimental record the user has filtered a diverse range of feeds in a natural way and potentially aided the system in making connections between inputs and outputs.

### Robots as assistants for semantic authoring

Robots might also play the role of the "nudging" system described above to assist users to use appropriate vocabularies. Again the user can select a Robot that is appropriate to their research domain or the specifics of their experiment. These Robots could take at least two forms. Where a structured description or minimal information requirement exists for a specific type of experiment a Robot could parse that description to generate a form for the user to fill out. Such functionality could also be achieved through the direct interpretation of structured description formats into web forms but Wave has the potential to bring this functionality directly to where the user is working, in their inbox.

More exciting though is the potential for the Wave framework to enable the contextual markup and structuring of *free *text *as the user is typing*. Wave contains a powerful contextual spell checker and language translation engine that combines real time monitoring of user's typing and inline insertion of corrected words or a parallel translation into another language. This is based on a large database of phrase fragments. It is possible to imagine a similar database of research domain specific fragments, offering specific forms of words or terms to the user as they type, and inserting annotation and links to specific web based objects. Once the user agrees a specific term the Robot could generate a feed of the marked up and annotated relationships within the experimental record.

### Robots as feed and record generators

With the idea that Robots can generate feeds we can see that Wave has the potential to act as a central coordinating space for incoming and outgoing feeds that are selected from a wide range of options by the user, or indeed by other Robots acting in an automated fashion based on actions of the user. Our "feed of relationships" can be generated by Robots recording the actions they have performed or monitoring the actions of other Robots or users. The record of what actions took place, when they occurred and who triggered them is recorded in the Wave itself via the versioning system. Finally a "script" that would enable a third party to repeat at least the digital processes that are recorded within the wave can be created either manually or automatically by simply creating a copy of the Wave without input data. The Robots remain as participants, poised to take action when the data or connections are added back in.

Wave or the concepts behind it therefore has the potential to provide a system in which the requirements identified previously: versioning, feed wrangling, and flexible semantic annotation, can be developed. It is important to note that much of this *could *be done with currently existing tools. Such a system could be put together using PHP, structured descriptions of vocabularies, intelligent sample management systems, and high quality back end data records. However such systems would be specific, taking the user away from their existing authoring and communication systems. What Wave provides is a framework in which all of the tooling that makes these systems easy to develop is already provided in a communication and authoring tool that could be their default environment. The future penetration and effectiveness of the Wave protocol remains to be seen but with Google behind it and it being an open federated protocol the prospects are positive. If Wave itself does not gain widespread use then we can expect many of the ideas and design principles behind it will become part of the everyday web over the next few years.

## Provenance and Security

A major concern with a distributed model of the research record is security and provenance. Security issues are raised both because valuable content is distributed amongst systems that are not under the institution's control and therefore may be lost due to technical or financial failure, but also because valuable intellectual property is potentially wandering free on the open web. On top of this there are concerns about the reliability of provenance records; who deposited a specific piece of data or document, and when did they do so? Third party date stamps and identifiers might, at a naive level, seem a good record of priority but the standards and reliability of these third party services is rarely validated to any specific standard. In terms of provenance trails, how a specific statement or datafile has been arrived at, existing consumer focused tools are generally very poor at assisting the user in describing what derivative material they have used. Slideshare, while supporting Creative Commons licensing of slidesets does not provide any easy means of describing where a given slide or image derives from. Given that it is only recently that an intellectual property case has been concluded with a contribution from an electronic laboratory notebook, and the lack of widely accepted standards for maintaining and describing security and provenance on the web it will not surprising if corporate users in particular are unconvinced by the idea of a distributed and openly available model of their research records.

There are three main answers to such a criticism; the most water tight from a security perspective is that the whole network can be maintained behind a corporate firewall. Thus an internal community has access to all the functionality provided by the networked record but the content is "safe" from outside eyes. Such an arrangement loses the value of being wired into the wider information infrastructure of the public web but for large enough companies this may be acceptable. Hybrid inside/outside systems may be feasible but are likely to be porous, especially given the weak point in any security system is almost always the people who use it. Nonetheless there is value in creating shareable *references *to objects via URLs even if those objects themselves are not shared or accessible. There are a range of partial models that may be valuable in specific contexts.

The second possible answer to criticism is that we need to develop the tool set and standards that lets us describe and validate security and provenance of objects on the web. Distributed access and authentication control is an active area of research development with tools such as Shibboleth [21], OpenID [22], and OAuth [23] providing mechanisms for authentication and access control targeted at the consumer web. A wide range of developing offerings to support identity management for researchers is also developing utilising various underlying protocols. At one level distribution should make the description of resources more reliable. Where multiple services and multiple people point at resources the level of possible trust should be much greater than when one or two, often internal, signing authorities are being relied on. This distributed network of date stamps and comments while more diffuse is actually much more difficult to fake as it requires multiple different records, often created by different people, on different services and servers to be changed. It is also however more difficult to aggregate and document such a distributed record in a way that makes sense in the context of today's record keeping systems and legal records.

The final answer is to say that if you're attempting to build a collaborative research record that leverages networks and the web and you are focussed on protecting your content from other people then you are missing the point. The entire purpose of the exercise is to get the most out the information and the people manipulating it on the web by providing content that they can re-use, and in the process, pass useful information and connections back to you. If you do this in a partial way then you only get a partial benefit. It is only by allowing people outside your organization to wire your samples, data, and ideas into the wider linked open web that you gain benefits over and above what you can gain by simply using your own internal expertise more effectively. For very large companies an entirely internal web may be a viable strategy but for most small and medium companies or public research organizations the gains are potentially much greater than the losses. This doesn't mean there is a free for all. Provenance and identity are also important in this view, but here it is less about demanding that credit be assigned and more about gaining the most from connections that other people make by understanding who they are and where they are coming from.

Again the Wave protocol offers some interesting potential solutions to these problems. Parts of a given wave can have different participant lists and even be hosted on different servers. It is possible for two researchers from Company A to have a private conversation within a wave for which the root is on a public server hosted by Organization B without any of the other participants being able to see their conversation. In fact, the private conversation never needs to leaves the Company A Wave Server and can stay behind the corporate firewall. This distribution of the actual content between secure and unsecure servers can be combined with existing fine grained approaches to XML document access [24,25] may have significant implications for how we think about the management of valuable documents and objects.

Waves current contain a full versioned record of changes enabling credit to be assigned to various contributions. There is also the suggestion that the full power of code version repositories will be applied to tracking of Waves, both within the history of a single wave, but also when new waves are spawned from existing waves, tracking their antecedents and what was passed and when, as well as enabling "fork" and "merge" operations of multiple independent versions of the same wave.

Whether or not Wave offers a technical solution the problems of attribution, provenance, and access rights are general ones that are facing the consumer web as users gradually gain a more sophisticated understanding of what privacy means in a networked world. If the view is accepted, that in wiring the research record into the wider web that the benefits gained outweigh the losses, then it follows that issues of access control with respect to viewing objects are less important. However being able to reliably identify the author of a dataset or other object becomes much more important in determining how you will respond to the way they have interacted with your materials. Therefore provenance becomes a key issue and reliable authentication mechanisms that provide strong identities are crucial. There will no doubt be much development over the next few years of mechanisms for tracking the network of citations between objects on the web and using this to assign priority and precedence of ideas and statements. All of these will improve the toolset available to work with distributed research records of the type being discussed here.

## Conclusion

The web and its organization fundamentally challenges the idea of the research record as a single document or record and removes the constraints created by a physical record or document to enable a multifaceted, multifunctional, and multimedia laboratory notebook. The web itself has evolved from its original form of linked static documents to dynamic services and sites populated by objects created by their users and content automatically aggregated and re-published in new contexts and new forms. The vision of the web as a dynamic network of addressable objects and their relationships can be traced right to its earliest origins in the early 1990s but it is only now being realized.

Fully exploiting the infrastructure and functionality of the web to create a web-native laboratory record requires re-thinking the traditional view of the laboratory notebook as a linear narrative of events. By creating individual addressable objects that refer to physical samples and materials, laboratory or computational processes, and data files, it is possible to create a dynamic record that can be directly linked into the growing semantic and linked data web. The web native laboratory notebook is a combination of the web of data and the web of things. If the best available services are used for each kind of object then the actual laboratory record can be reduced to a feed describing the relationships between these objects and functionality that has been specifically designed around those objects can be fully exploited.

Such an approach recognizes that the processes that occur in science, particularly in an experimental laboratory, are often haphazard. The key to *capturing *the research record, the issue we are focussed on here, is to ensure that the objects created during the research process are recognized, stored and indexed, and to make it as easy as possible for the researcher to record the relationships between these objects. The use of purpose built services for specific object types is intended to make their capture and "wiring up" as straightforward as possible. This distribution does not need to be seen as antithetical to integrative and aggregation approaches to capturing and describing knowledge [17]. Rather it can be thought of as an efficient way of generating and indexing the raw material, the data and information, that will make up these "datuments" to facilitate their production.

The beauty of this approach is that it doesn't require users to shift from the applications and services that they are already using, like, and understand. What it does require is intelligent and specific repositories for the objects they generate that know enough about the object type to provide useful information and context. What it also requires is good plugins, applications, and services to help people generate the lab record feed. It also requires a minimal and arbitrarily extensible way of describing the relationships. This could be as simple html links with tagging of the objects (once you know an object is a sample and it is linked to a procedure you know a lot about what is going on) but there is a logic in having a minimal vocabulary that describes relationships (what you don't know explicitly in the tagging version is whether the sample is an input or an output). But it can also be fully semantic if that is what people want. And while the loosely tagged material won't be easily and tightly coupled to the fully semantic material the connections will at least be there. A combination of both is not perfect, but it's a step on the way towards the global data graph.

The technical challenges of implementing this vision are formidable. Authoring tools are needed, along with well designed repositories; a whole infrastructure of services and tools for pushing information between them. In this context the announcement of the Google Wave protocol is a very interesting development. The functionality that is described has an enormous potential to make the implementation of many of these tools much easier. The proof of this will be in the development of useful functionality within this platform. Google has the brand awareness and expertise to make such a revolution in online communication technology possible. They have set the agenda and it will be a very interesting story to follow.

The view of the laboratory record as a distributed set of objects on the open web is closely linked with the agenda of the Open Research Movement. The presumption is that the gains made by wiring your own ideas, samples, and results into the wider information graph, far outweigh the losses. Only limited gains could be made by adopting this architecture but keeping the content closed off from the wider web. Thus its adoption and development depends closely on the users view of the future of scientific communication. If you accept the vision of open communication and the idea that it will make your own research more competitive then this is a path to follow. The web was built for the sharing of data amongst research scientists. We are only just learning how to do that effectively and efficiently.

## Competing interests

The author declares no competing interests.
